# Theoretical and experimental studies on the oil-based emulsion spray

**DOI:** 10.3389/fpls.2023.1164200

**Published:** 2023-04-19

**Authors:** Chen Gong, Fujun Chen, Can Kang

**Affiliations:** ^1^ School of Agricultural Engineering, Jiangsu University, Zhenjiang, China; ^2^ School of Energy and Power Engineering, Jiangsu University, Zhenjiang, China

**Keywords:** oil-based emulsion spray, theoretical model, sheet structure, image processing, nozzle configuration

## Abstract

Oil-based emulsion is a common herbicide formulation in agricultural spray, and its atomization mechanism is different from that of water spray. In this paper, a theoretical model based on the characteristics of spray sheets was proposed to predict the spray droplet size for oil-based emulsion spray. An image processing method was used to measure droplet size distributions for different spray pressures and nozzle configurations, and the measured results were used to validate the theoretical model. The results show that oil-based emulsion spray is characterized by the web structure constituted by perforations. The liquid originally occupied by spray sheets eventually gathers in these web structures. The proposed theoretical model is based on the size of the nozzle exit, the angle of spray sheets, and the perforation number in the web structure, which are relatively easy to obtain. The theoretical droplet size is in inverse proportion to the square root of the perforation number in the web structure while in proportion to the square root of the area of the nozzle exit. The captured images of spray sheets and the measured droplet size distribution show consistency with the theoretical prediction. The difference between theoretical results and measured volumetric median diameter is less than 10% for different spray pressures and nozzles.

## 1 Introduction

The flat-fan spray is a commonly used method to deliver agricultural chemicals and control weeds in agricultural production (Kalsing et al., 2018; Qin et al., 2021; Gong et al., 2022). The spray characteristics of the flat-fan nozzle have been widely studied; however, most of them are focused on experimental measurement of droplet characteristics (Ellis et al., 2021; Perine et al., 2021; Szarka et al., 2021). Theoretical research for the flat-fan spray, especially as agricultural chemicals are used, is limited.

Different from the cylindrical liquid jet, the fat-fan spray has a complex geometry that makes it difficult to find simplified assumptions (Lefebvre, 1989). Nonetheless, there have been some attempts to establish a theoretical model to describe the atomization process and forecast the droplet size of the flat-fan spray (Kashani et al., 2018; Asgarian et al., 2020). For water spray, the most common theoretical model is termed the “wave model”, which was first proposed by Fraser et al. (1962). This model has been widely studied and improved until recent years (Kooij et al., 2018; Post and Hewitt, 2018). The wave model has featured a wave-like structure, which is supposed to be caused by the velocity difference between gas and liquid at the interface (Shao et al., 2018). These wave-like structures are amplified by the Rayleigh–Taylor destabilization, and it introduces the thickness modulations of a liquid sheet (Kooij et al., 2018). It eventually leads to the breakup of the liquid sheet as the local thickness is thin enough. For water spray, the wave model is validated by good prediction of the droplet size (Post and Hewitt, 2018). However, the wave model may be not suitable, as agricultural chemicals are used. Many works indicated that the droplet characteristics of oil-based emulsion spray are significantly different from those of water spray even when the percentage of active ingredients of the chemical in the spraying liquid is only 0.1% (Lin et al., 2016; Carvalho et al., 2017).

Oil-based emulsion is a common herbicide formulation that has an obviously different atomization process from that associated with water spray (Gong et al., 2020; Gong et al., 2021). In general, the perforations in the liquid sheet are supposed to be the feature structure of an oil-based emulsion spray (Hilz, 2013; Vernay, 2015). It is argued that the generation of perforation has a close relationship with the characteristics of oil droplets in the oil-based emulsion solution (Hilz et al., 2012; Cryer and Altieri, 2017; Cryer et al., 2021). Once perforations are generated, they will expand due to the surface tension (Vernay et al., 2017). With the development of the perforations, they will eventually meet each other, and their borders will coalesce into ligaments (Li et al., 2021). The perforations are supposed to cause an early breakup of the liquid sheet; therefore, oil-based emulsions have larger droplet sizes compared with those of water spray (Carvalho et al., 2017; Alves et al., 2018). Meanwhile, perforations in the spray sheets can balance the pressure difference between the two sides of the liquid sheet; therefore, the amplitude of wave-like structures is suppressed (Gong et al., 2020). As a result, less loss of kinetic energy is caused by the flapping of the spray sheet, and spray droplets have higher velocity when compared with water spray (Cloeter et al., 2010; Hilz and Vermeer, 2013). Based on the review above, it can be concluded that oil-based emulsion spray has an obviously different atomization process and feature structure from those of water spray. Research related to oil-based emulsion spray is limited; as far as we know, only Altieri and their colleagues have made some attempts to establish a theoretical model to describe the atomization process (Altieri et al., 2014; Altieri and Cryer, 2018). Their models are based on the development of perforations; therefore, the position and diameter of the primary perforations are of vital importance. However, as indicated by Altieri and Cryer (2018), these parameters are difficult to obtain since the mechanism of perforation nucleation is unknown. Undoubtedly, these works provide inspiration for further research.

In this paper, a theoretical model based on web structure was proposed to predict the droplet size of an oil-based emulsion spray. The number of perforations in the web structure and spray droplet sizes of the primary breakup were measured based on image processing methods. The theoretical and experimental results were compared and discussed for different spray pressures and nozzle configurations.

## 2 Materials and methods

### 2.1 Capture of spray sheet

**Figure 1:**
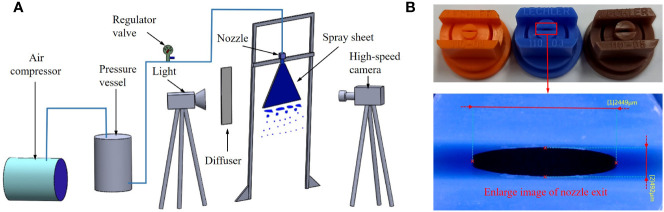
**(A)** Schematic view of experimental apparatus. **(B)** Nozzle image and enlarged image of nozzle exit.

**Figure 2:**
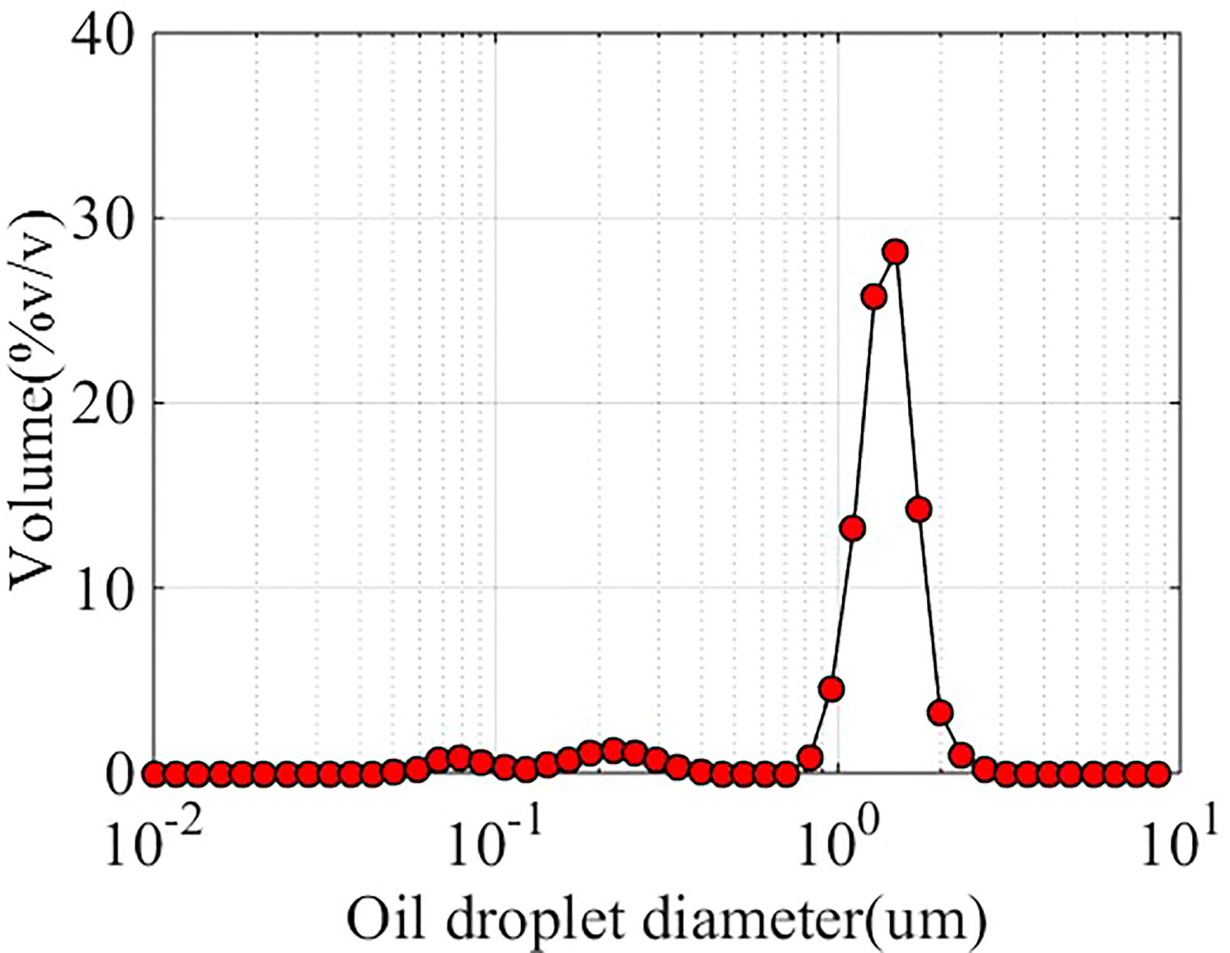
Size distribution of oil droplet in oil-based emulsion solution.

The physical information of spray sheets is the foundation of a theoretical model. Therefore, as shown in **Figure 1A**, an experimental system was built to capture the liquid sheet of oil-based emulsion spray. The oil-based emulsion solutions were prepared and stored in the pressure vessel and pressured by an air compressor. The oil-in-water emulsion solution was prepared with water and commercial butachlor (Jiangsu Lvlilai Co., Kunshan, Jiangsu, China). The main composition is as follows: 60% w/w butachlor, 15% w/w cyclohexanone (dissolvant), 9% w/w styrylphenyl polyoxyethylene ether (emulsifiers), and 6% w/w sodium alkylbenzene sulfonate (emulsifiers). The volumetric concentration of the commercial butachlor in the solution is 0.1%. The size distribution of oil droplets in an oil-based emulsion solution was measured using the Malvern particle size analyzer (type: Zetasizer Nano ZS90), and the result is shown in **Figure 2**. A pressure valve (SMC AR-3000, SMC China Co., China; accuracy of 0.02 MPa) was used to regulate the spray pressure. Three spray pressures, namely, 0.1, 0.3, and 0.5 MPa, were selected in the experiments. After the pressurized liquid passes through the nozzle, a spray is produced. Three standard flat-fan nozzles, ST-110-01, ST-110-03, and ST-110-05 (Lechler Inc., Germany), were used in the experiments. As shown in **Figure 1B**, the long diameter (*N_L_*) and shorter diameter (*N_s_*) If the discharge orifice of the nozzles were measured using a microscope (VHX-900F, Keyence Co., Japan) with a magnification factor of 50, and the measured results are listed in **Table 1**.

**Table 1:** Structural parameters of different nozzle exits.

	Long diameter *N_L_*/μm	Short diameter *N_s_*/μm
ST-110-01	1,450	242
ST-110-03	2,449	492
ST-110-05	2,944	695

The spray sheets and droplets were captured using an Olympus i-Speed TR high-speed camera and a macro lens (Tokina Macro 100 F 2.8 D, Olympus Co., Japan). A backlit-imaging arrangement was adopted, and a diffuser was mounted between the light source and the spray sheets to ensure a uniform light distribution over the monitored plane. The exposure time of the camera was set to 2.16 μs, and the frame rate was set to 2,000 fps (frame per second). The resolution of the captured images was 1,280 × 1,024 pixels.

### 2.2 Measurement of spray sheet

**Figure 3:**
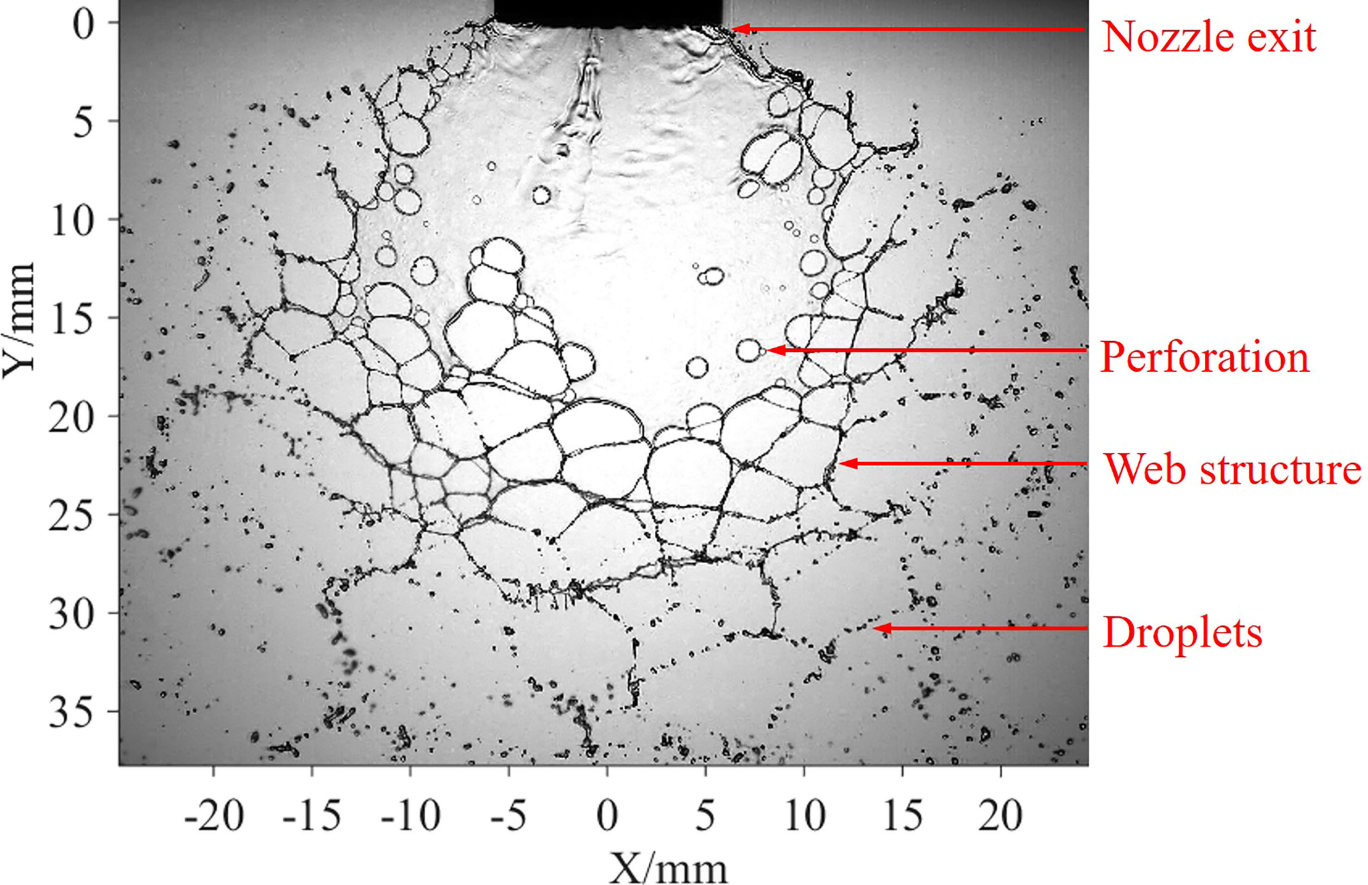
Sheet structure of oil-based emulsion spray.

A typical image of the sheet structure of an oil-based emulsion spray is presented in **Figure 3**. After the spray liquid leaves the nozzle exit, it first forms a flat-fan sheet, and then perforations are generated in the sheet. With the development of perforations, they contact each other and form a web-like structure. Eventually, these web structures break up into ligaments and droplets.

**Figure 4:**
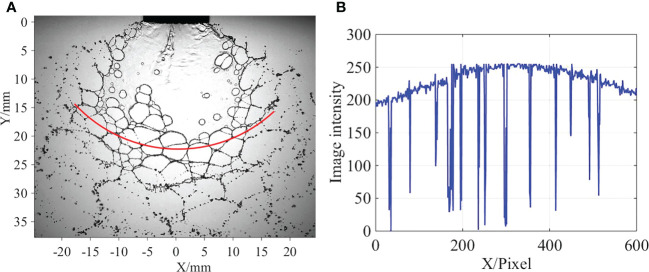
Measurement of perforation number in web structure. **(A)** Red line indicates the positions where image intensities of pixels were measured. **(B)** Variation of image intensity.

An image processing method was proposed to measure the perforation number in web structures. Images are constructed based on digital information representing image intensities. The digital information essentially reflects the morphological characteristics of the flow structures. In the spray image, the image intensities of the liquid and the background air are different. Based on this principle, the edges of web structures and air can be identified by processing the image intensities. As indicated in **Figure 4A**, at the radial distance where the web structure is located, a red line is plotted along the circular arc. The image intensities of the pixels on this red line were measured through a commercial image analysis code Image Pro Plus, and the results are plotted in **Figure 4B**. Due to reflection and refraction, the pixels of the liquid show low image intensities relative to those of the air. Therefore, as indicated in **Figure 4**, once the red line passes through an edge of the web structure, there will be a trough of wave on the curve of image intensity. Based on the commercial code MATLAB (Cai, 2020), a digital signal processing method was designed to count the number of wave troughs; therefore, perforation numbers in web structure can be obtained. Under the same experimental condition, 20 images were processed. The average result obtained based on these 20 images was obtained to determine the perforation number.

### 2.3 Measurement of droplet size

**Figure 5:**
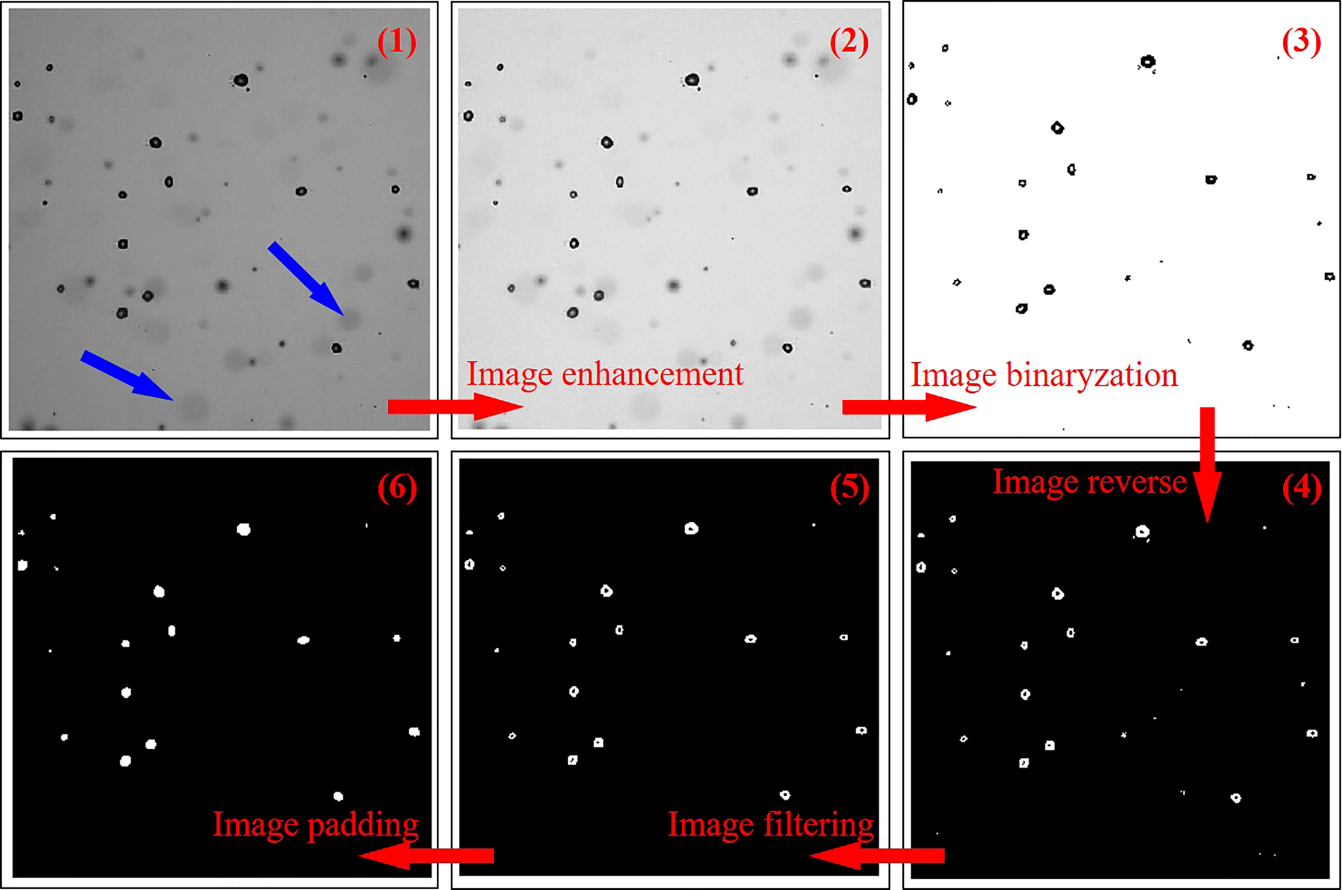
Image processing method.

Light transmittance of the oil-based emulsion solution is different from that of water; therefore, the droplet size measurement based on light diffusion or refraction may cause errors (Sijs et al., 2021). Here, we measure droplet sizes based on image processing. In order to measure the primary diameter of the spray droplet, the position of the sampling window is set as close as possible to the breakup position of the spray sheet. The size of the sampling window is 10 × 10 mm. A typical raw image within a sampling window is shown in **Figure 5**(1); spray droplets distribute in different depths of the field. The droplets that are not on the focal plane (indicated with blue arrows) should be filtered. In order to remove those noises, the initial raw image was enhanced using the Retinex algorithm, and then the Otsu algorithm was selected to determine the image intensity threshold and achieve a binarization image. After that, the binarization image was reversed, then the noise was filtered, and the image was padded. Eventually, the pre-processed image of spray droplets was obtained (**Figure 5**(6)). The boundary of the spray droplets was detected and extracted using the Bwlabel function, and then the droplets were measured with the Regionprops function (Cai, 2020). In order to ensure enough samples and reduce random error, multiple images of different times were processed and measured. In this paper, the number of samples (spray droplets) is more than 5,000 for each experimental condition.

## 3 Results and discussion

### 3.1 Theoretical model

**Figure 6:**
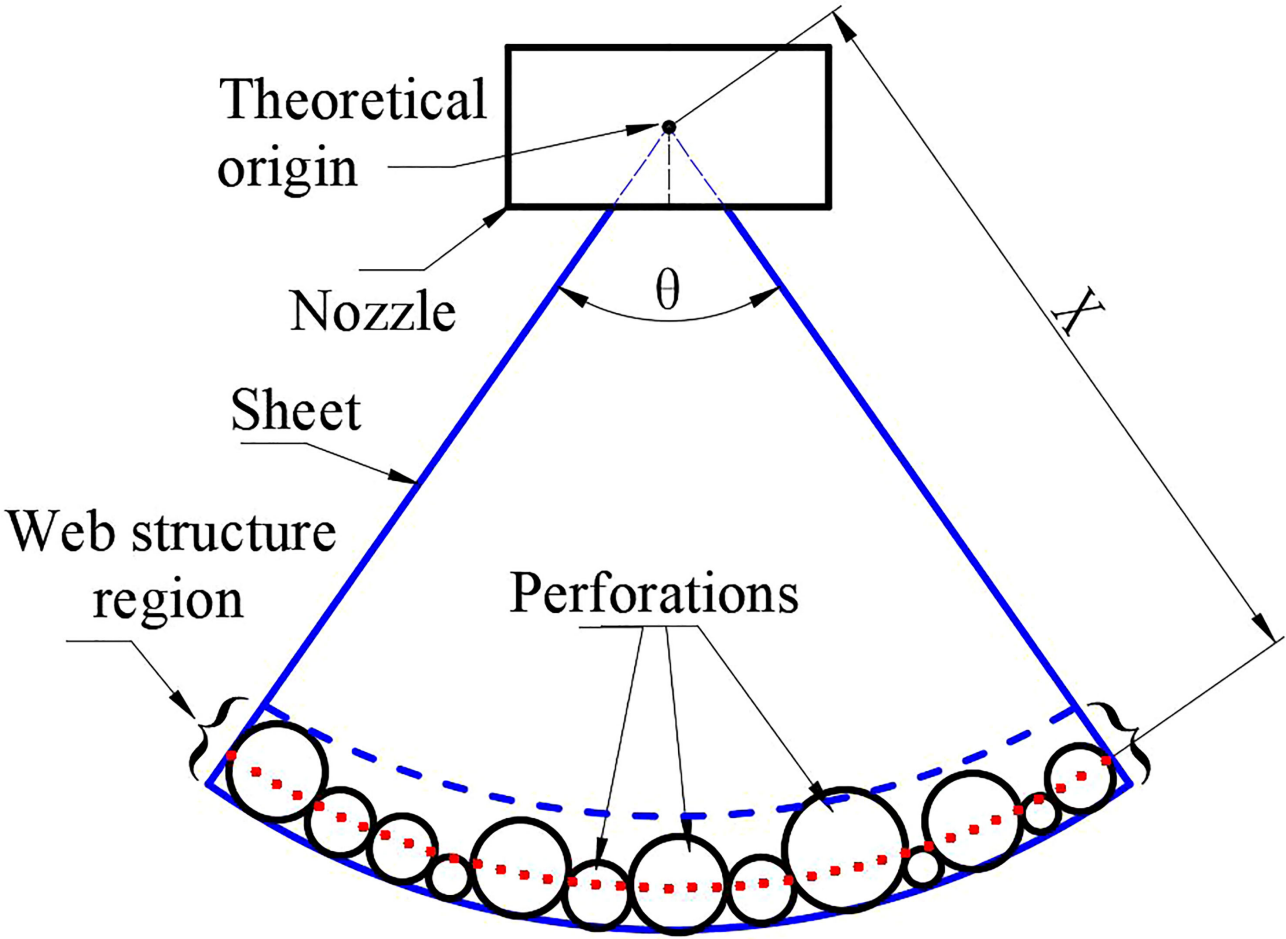
Theoretical schematic view of oil-based emulsion spray.

Based on the image of the spray sheets shown in **Figure 3**, it is found that the web structures are the final structures prior to the breakup of the liquid sheet. Therefore, it can be inferred that all the liquid occupied by the sheet will finally gather at the edges of the web structures. Furthermore, the diameter of the edges determines the initial size of ligaments and droplets. We proposed a theoretical model that is based on web structure to forecast droplet sizes. The sheet structure is simplified and plotted in **Figure 6**. The point of intersection of the sheet edges is defined as the theoretical origin. The circles are used to represent the perforations, and the web structure region consists of multiple perforations with different diameters. The average position of the centers of these circles is indicated with a red dash curve, as shown in **Figure 6**.

The original liquid volume of the web structure region is defined as *V*_1_,


(1) $$V_{1}=\bar{L}\;\bar{W}\;\bar{\tau },$$

where $\bar{W}$ , $\bar{L}$ and $\bar{\tau }$ denote the average width, the average length, and the average sheet thickness, respectively, of the web structure region.

Here, the arc length of the red dash curve was used to represent the average length of the web structure region and is expressed by the following:


(2) $$\bar{L}\;=\frac{\pi X\theta }{180},$$

where *X* represents the radial distance between the red dash curve and the theoretical origin. *θ* denotes the angle of the spray sheet.

The average diameter of the perforations was used to represent the average width of the web structure region:


(3) $$\bar{W}\;=\frac{D_{1}+D_{2}\ldots +D_{n}}{N}$$

where *D*_1_, *D*_2_… *D_n_* denote the diameters of the perforations. *N* denotes the number of perforations.

The perforations are tangent; therefore,


(4) $$D_{1}+D_{2}\ldots +D_{n}=\bar{L}.$$

The sheet thickness decreases as the sheet moves away from the nozzle. According to Altieri et al. (2014) and Fraser et al. (1962), there is a relationship between sheet thickness and radial distance:


(5) $$\tau =\frac{\kappa }{X},$$

where *k* is the spray parameter. It is related to the nozzle dimension and spray angle:


(6) $$k=\frac{N_{L}\tau_{n}}{2\text{sin}\left(\theta /2\right)},$$

where *N_L_* is the long diameter of the nozzle exit and *τ_n_* is the sheet thickness at the nozzle exit, which is equal to the short diameter *N_s_* if the nozzle exit.

Combining Eqs. 1–6 yields


(7) $$V_{1}=\frac{\pi X\theta }{180}\;\frac{\pi X\theta }{180N}\frac{N_{L}N_{S}}{2X\text{sin}\left(\theta /2\right)}=\frac{\pi^{2}\theta^{2}N_{L}N_{S}X}{64,800N\text{sin}\left(\theta /2\right)},$$

The liquid volume, *V*_2_, in the edges of the web structures is defined as follows:


(8) $$V_{2}=\bar{S}L_{\text{s}},$$

where $\bar{S}$ denotes the average cross-section area of edges of the web structure and *L_s_* denotes the total length of edges of the web structure.

Suppose the edges of the web structure have circular cross sections:


(9) $$S=\pi R_{s}^{2},$$

where *R_s_* denotes the average radius of circular cross sections.

The total length of the edges of the web structures is equal to half the total perimeter of the perforations since two perforations share one edge.


(10) $$L_{s}=0.5\pi \left(D_{1}+D_{2}\ldots +D_{n}\right).$$

Combining Eqs. 8–10 yields


(11) $$V_{2}=\pi R_{s}^{2}\;0.5\pi \left(D_{1}+D_{2}\ldots +D_{n}\right)=0.5\pi^{2}R_{s}^{2}\frac{\pi X\theta }{180}.$$

Assume that all the liquid that was originally contained in the perforations now resides in the edges of web structures. Based on mass conservation,


(12) $$\rho V_{1}=\rho \frac{\pi^{2}\theta^{2}N_{L}N_{S}X}{64,800N\text{si}n\left(\theta /2\right)}=\rho 0.5\pi^{2}R_{s}^{2}\frac{\pi X\theta }{180}=\rho V_{2},$$

where *ρ* denotes the density of spray liquid.

Based on Eq. 12, the diameter of the edge of the web structure can be calculated as follows:


(13) $$D_{s}=2R_{s}=2\sqrt{\frac{\theta N_{\text{L}}N_{\text{S}}}{180\pi N\text{sin}\left(\text{θ}/2\right)}}.$$

The image of the spray sheets indicates that the diameter of the primary droplets has a similar size to the edge of the web structure. Therefore, *D_s_* is used to characterize the primary diameter of spray droplets.

### 3.2 Comparison with experimental results

From Eq. 13, it can be deduced that the theoretical diameter is determined by the size of the nozzle exit, the angle of spray sheets, and the number of perforations in the web structure. These parameters have a close relationship with nozzle configuration and spray pressure. Therefore, the theoretical results for different spray pressures and nozzle configurations were estimated and validated through measured results.

**Figure 7:**
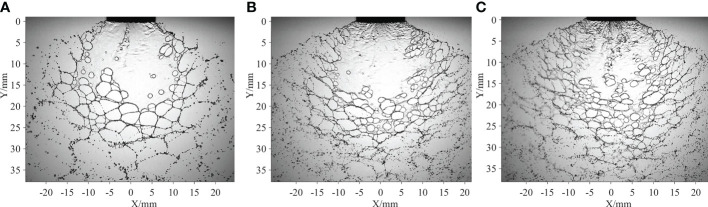
Spray sheets at different spray pressures. The spray sheets are produced by the ST-110-03 nozzle, and the volumetric concentration of the oil-based emulsion is 0.1%. **(A)** *P*=0.1 MPa **(B)** *P*=0.3 MPa **(C)** *P*=0.5 MPa.

First, the effects of the spray pressure are discussed. Three typical spray sheets obtained at different spray pressures are presented in **Figure 7**. For the angle of the spray sheet, it increases with spray pressure; however, the increasing ratio decreases. After the spray liquid is ejected from the nozzle, the liquid sheet expands along a spanwise direction due to the configuration of the flat-fan nozzle exit. The higher the spray pressure, the higher the kinetic energy, and the larger the angle of the spray sheet. Meanwhile, limited by the nozzle configuration, the increase of the angle of the spray sheets attenuates as it attains a critical value. For the perforations in the web structure, the number increases, but the average size decreases with increasing spray pressure. It is proposed that the vortices and rollup motion may thus excite the movement of liquid at different positions, causing the oil droplets in the emulsion to interact with each other, and larger oil droplets are thereby produced (Gong et al., 2020). Large oil droplets are more likely to generate perforations (Cryer and Altieri, 2017); therefore, the number of perforations increases with spray pressure. Due to the increase in the perforation number, perforations come into contact much more easily. Consequently, the development of the perforations will be resisted by adjacent perforations. Therefore, the average size of the perforations in the web structure decreases.

Based on the measured angle of the spray sheets and the number of perforations in the web structure, the theoretical results are estimated and presented in **Table 2**. The theoretical values decrease with the spray pressure. Thin spray sheets and small perforations are responsible for the decrease in theoretical data. The liquid at the edges of the web structure is supposed to come from the liquid, which was originally contained in the perforations (Fraser et al., 1962). Alternatively, the area of the perforation and the thickness of local spray sheets where the perforation is located determine the size of the edges of the web structure. As shown in **Figure 7**, the average size of perforations decreases with spray pressure. Meanwhile, the area of the spray sheets increases with spray pressure; therefore, the thickness of the spray sheets decreases. The theoretical data are in accordance with the experimental results.

**Table 2:** Theoretical results at different spray pressures.

Spray pressure (*P*/MPa)	Measured angle of the spray sheets (*θ)*	Measured number of perforations (*N)*	Estimated theoretical diameter (*D_s_/*um)
0.1	119.68 ± 1.49	19.6 ± 1.90	237.80
0.3	127.10 ± 2.34	29.7 ± 2.45	195.63
0.5	129.84 ± 1.60	42.6 ± 4.21	164.15

**Figure 8:**
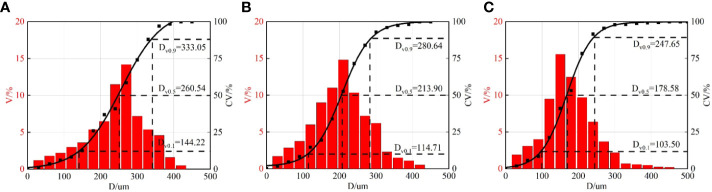
Droplet distributions at different spray pressures. V% denotes percentage of volume of droplets of different sizes. CV% denotes cumulative volume of droplets. **(A)** 0.1 MPa **(B)** 0.3 MPa **(C)** 0.5 MPa.

The spray droplet distributions measured with the developed image processing method are presented in **Figure 8**. With the increase of the spray pressure, the distribution of droplets moves from a large size zone to a smaller size zone. The volumetric droplet size metrics *D_V10_*, *D_V_*_50_, and *D_V90_* also decrease with spray pressure. The theoretical results are close to the volumetric median diameter *D_V_*_50_. The difference between theoretical results and the measured volumetric median diameters is less than 10% for different spray pressures. The volumetric median diameter *D_V_*_50_ is commonly used to characterize the general size of spray droplets in agriculture (Miranda-Fuentes et al., 2018). It is indicated that the theoretical model can well predict the droplet size of oil-based emulsion spray at different spray pressures.

**Figure 9:**
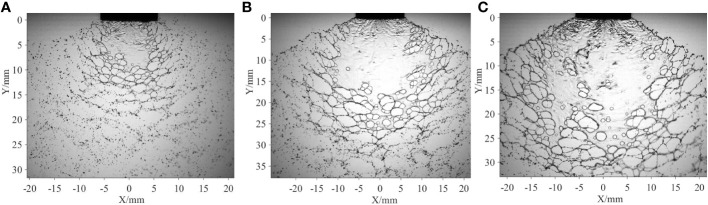
Spray sheets for different nozzles. The spray pressure is 0.3 MPa, and the volumetric concentration of the oil-based emulsion is 0.1%. **(A)** ST-110-01 **(B)** ST-110-03 **(C)** ST-110-05.

Three typical spray sheets for different nozzle configurations are presented in **Figure 9**. The nozzle configuration has a significant effect on the spray sheet. Typically, for the ST-110-01 nozzle, the spray sheets break up earlier in both streamwise and spanwise directions. The spray sheets have no apparent boundary; therefore, the angle of the spray sheets was estimated based on the trajectory of spray droplets at the two edges of the spray sheet. Generally, the angles of the spray sheets discharged from the three nozzles have no significant difference. However, the area of spray sheets obviously increases with the size of the nozzle exit. The thickness of the primary spray sheets is determined by the size of the nozzle exit, and the spray sheets associated with the ST-110-01 nozzle have a relatively small thickness. Therefore, the oil droplets in the spray liquid are much easier to reach the interface of spray sheets (Cryer and Altieri, 2017). As a result, the perforations are generated at the positions close to the nozzle exit. Accordingly, the spray sheets break up earlier, and the area is small. For the perforations in the web structure, the number increases with the size of the nozzle exit. A possible reason is that the flow rate increases with the size of the nozzle exit, more oil droplets are contained in the spray sheets, and the probability that oil droplets interact with each other and the formation of larger droplets is high. Large oil droplets are efficient in terms of causing perforations (Vernay, 2015). Accordingly, the spray sheets of the ST-110-05 nozzle involve more perforations.

The measured angle of the spray sheet, the number of perforations in the web structure, and the theoretical results for different nozzles are presented in **Table 3**. Theoretical values increase with the size of the nozzle exit. The diameter of ligaments is supposed to have a close relationship with the thickness of spray sheets at the position where the sheet breaks up. The sheet thickness is in proportion to the area of the nozzle exit while in inverse proportion to the radial distance (Altieri et al., 2014). From **Figure 9**, it can be inferred that the breakup length of the spray sheets of the ST-110-01 nozzle is about half of that of the ST-110-03 nozzle and one-third of that of the ST-110-05 nozzle. However, based on the measured results shown in **Table 1**, the area of the nozzle exit of the ST-110-03 nozzle is three times more than that of the ST-110-01 nozzle, while the area of the nozzle exit of the ST-110-05 nozzle is nearly six times that of the ST-110-01 nozzle. Therefore, the ST-110-05 nozzle has a larger theoretical diameter.

**Table 3:** Theoretical results at different nozzle configurations.

Nozzles	Measured angle of the spray sheets *(θ)*	Measured number of perforations (*N)*	Estimated theoretical diameter (*D_s_/*um)
ST-110-01	121.69 ± 1.90	18.9 ± 1.65	135.31
ST-110-03	127.10 ± 2.34	29.7 ± 2.45	195.63
ST-110-05	126.25 ± 2.00	38.2 ± 3.02	231.63

**Figure 10:**
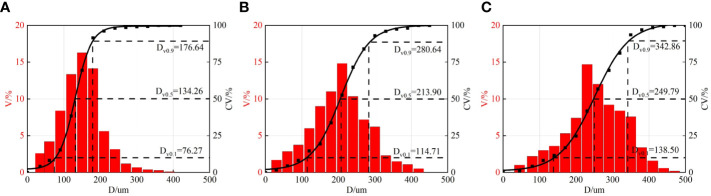
Droplet distributions for different nozzles. V% denotes percentage of volume of droplets of different sizes. CV% denotes cumulative volume of droplets. **(A)** ST-110-01 **(B)** ST-110-03 **(C)** ST-110-05.

Similarly, the spray droplet distributions for different nozzles are measured and presented in **Figure 10**. In general, the experimental results are in accordance with the theoretical prediction. Furthermore, the theoretical results are close to the volumetric median diameters. The maximum difference between theoretical and experimental results is less than 10% for different nozzles. The difference might be due to the simplification of the spray structure. The structure of spray sheets varies with time; some simplifications are needed so as to establish a theoretical model. In our model, circles are used to represent the perforations, and spray droplets are assumed to have a spherical shape. In practice, perforations have complex structures, and some of the spray droplets are not spherical, and these reasons might bring some errors.

## 4 Conclusions

A theoretical model was proposed to predict the droplet size of oil-based emulsion spray. The droplet size distributions under different experimental conditions were measured with a developed image processing method, and the measured results were used to validate the theoretical model. Major conclusions drawn from the study are as follows:

1) The web structure constituted by perforations is the typical structure before the breakup of the liquid sheet of oil-based emulsion spray. The liquid originally occupying the sheet eventually gathers at the edges of the web structures. The dimension of the edges of the web structures determines the primary size of ligaments and droplets produced with the breakup of the spray sheet.

2) The proposed theoretical model is based on the size of the nozzle exit, the angle of the spray sheets, and the perforation number in the web structure. These parameters are relatively convenient to obtain by measuring the nozzle and the spray sheet. The theoretical model indicates that the droplet size is in proportion to the square root of the area of the nozzle exit while in inverse proportion to the square root of the perforation number in the web structure.

3) The experimentally captured images of spray sheets for different spray pressures and nozzles show that the theoretical results are well consistent with the experimental results under different experimental conditions. The theoretical results are close to the volumetric median diameter of the measured results. The difference between theoretical and experimental results is less than 10% for different spray pressures and nozzles.

## Data availability statement

The original contributions presented in the study are included in the article/supplementary material. Further inquiries can be directed to the corresponding authors.

## Author contributions

CG: Methodology, Writing-Original draft preparation. FC: Image processing. CK: Conceptualization, Writing-Review and Editing. All authors contributed to the article and approved the submitted version.
